# A pharmaceutical-related molecules dataset for reversed-phase chromatography retention time prediction built on combining pH and gradient time conditions

**DOI:** 10.1016/j.dib.2022.108017

**Published:** 2022-03-04

**Authors:** Thomas Van Laethem, Priyanka Kumari, Philippe Hubert, Marianne Fillet, Pierre-Yves Sacré, Cédric Hubert

**Affiliations:** a Laboratory for the Analysis of Medicines, University of Liège (ULiege), CIRM, B36 Tower 4 (route 688 CHU) +3, Avenue Hippocrate, 15, Liège 4000, Belgium; b Laboratory of Pharmaceutical Analytical Chemistry, University of Liège (ULiege), CIRM, B36 Tower 4 (route 688 CHU) +2, Avenue Hippocrate, 15, Liège 4000, Belgium

**Keywords:** High performance liquid chromatography, Small pharmaceutical compounds, Reverse phase liquid chromatography, Quantitative structure retention relationship

## Abstract

There is a rising interest in the modeling and predicting of chromatographic retention. The progress towards more complex and comprehensive models emphasized the need for broad reliable datasets. The present dataset comprises small pharmaceutical compounds selected to cover a wide range in terms of physicochemical properties that are known to impact the retention in reversed-phase liquid chromatography. Moreover, this dataset was analyzed at five pH with two gradient slopes. It provides a reliable dataset with a diversity of conditions and compounds to support the building of new models. To enhance the robustness of the dataset, the compounds were injected individually, and each sequence of injections included a quality control sample. This unambiguous detection of each compound as well as a systematic analysis of a quality control sample ensured the quality of the reported retention times. Moreover, three different liquid chromatographic systems were used to increase the robustness of the dataset.

## Specifications Table

**Table:** 

Subject	Analytical Chemistry
Specific subject area	Liquid chromatography retention time modeling (QSRR)
Type of data	Tables
How data were acquired	Instruments: 1) Two Waters® Alliance 2695 with a UV-visible photodiode array detector 2996 module 2) One Waters® Alliance 2695 with a UV-visible photodiode array detector 2998 module and an evaporative light scattering detector 2424 module Equipment: 1) Column Waters® XSelect HSS T3 100 × 2.1 mm 3.5 μm Software: 1) Waters® Empower 3Pro FR5 SR5 (build 3471) 2) Microsoft® 365 - Excel
Data format	Raw and processed data
Description of data collection	Each compound was solubilised individually. The sample solution composed of only one compound were injected on an HPLC system using a C18 column with a gradient starting at 100% buffer and reaching 95% of MeOH. The pH of the buffer and the gradient time were the two experimental parameters.
Data source location	Institution: University of Liège (ULiege), CIRM, Laboratory of Pharmaceutical Analytical Chemistry City/Town/Region: Liège, Liège Country: Belgium Latitude and longitude for collected samples/data:50.5723391, 5.5643619
Data accessibility	Repository name: Mendeley Data Data identification number: 1) DOI: 10.17632/csm5gsmr5t.1 2) DOI: 10.17632/2w64h8pvkc.1 3) DOI: 10.17632/7v5p4gsh4z.1 Direct URL to data: 1) https://data.mendeley.com/datasets/csm5gsmr5t/ 2) https://data.mendeley.com/datasets/2w64h8pvkc/ 3) https://data.mendeley.com/datasets/7v5p4gsh4z/

## Value of the Data

• The data provided contribute to the need for reliable data presenting retention times collected from diversified small compounds in diversified chromatographic conditions.
• Publishing large databases collected at several experimental conditions may help other scientists develop new modeling approaches and more general models on a wider range of chromatographic conditions for the retention behavior.
• The dataset is based on a selection of compounds relevant for other chromatographic modes such as ionic (IC) and hydrophilic interaction (HILIC) modes as well as the executed reversed phase mode (RPLC). Its design make it possible for other scientists to use expand it.
• The dataset is developed with robustness and reliability in mind. The retention times were acquired on three different systems managed by a strict quality system to assess their performances periodically. In addition, analyses are performed together with quality control (QC) samples to assess the system suitability.

## 1 Data Description

Table 1 shows the composition of buffers used for the mobile phases.

**Table 1:** Composition of buffers.

pH	Composition
2.7	Formic acid
3.5	Ammonium formate and formic acid
5.0	Ammonium acetate and acetic acid
6.5	Ammonium bicarbonate and formic acid
8.0	Ammonium bicarbonate

Table 2 describes gradient parameters of methods.

**Table 2:** Chromatographic methods.

#	φ_start_	φ_end_	Δφ	Time	Slope
1	0	95	95	20	4.75
2	0	95	95	60	1.58
3	0	19	19	4	4.75
4	0	6.3	6.3	4	1.58

φ: percentage of organic modifier, Δφ: difference of organic modifier between the start and the end of the gradient.

Table 3 includes a description of each HPLC system.

**Table 3:** Description of HPLC systems.

ID	HPLC System	Detector
1	Waters Alliance 2695	UV-visible photodiode array detector 2996 module
2	Waters Alliance 2695	UV-visible photodiode array detector 2996 module
3	Waters Alliance 2695	UV-visible photodiode array detector 2998 module Evaporative light scattering detector 2424 module

Table 4 presents the average retention time (t_R_) and relative standard deviation (RSD) [%] of the quality control sample's compounds for each condition.

**Table 4:** Average retention time (t_R_) and relative standard deviation (RSD) [%] of the quality control sample's compounds for each condition.

		20 min		60 min	
	t_G_	t_R_ (min)	RSD (%)	t_R_ (min)	RSD (%)
pH 2.7	U	2.2	2.0	2.2	2.0
	C	8.1	3.2	10.4	2.4
	I	21.4	1.6	49	0.7

pH 3.5	U	2.2	2.5	2.2	2.8
	C	8.1	5.4	10.5	5.2
	I	21.3	2.5	48.8	1.5

pH 5.0	U	2.2	2.7	2.2	2.7
	C	8.1	5.1	10.5	4.8
	I	20.2	2.6	45.6	1.9

pH 6.5	U	2.2	2.3	2.2	2.1
	C	8.0	4.4	10.6	4.2
	I	18	2.9	39.1	2.0

pH 8.0	U	2.2	1.6	2.1	1.7
	C	8.1	3.4	10.6	3.0
	I	18	2	38.7	1.3

t_G_: gradient time, U: uracil, C: 3-cyanopyridine and I: ibuprofen. The number of values used to calculate the RSD varies for each condition (n_min_ = 23).

Table S1 describes the solubilisation and the dilution solutions of standard compounds.

All .arw files are the raw data exported as comma-separated values (CSV) from Empower 3 software. For the UVDAD signal, the first column is the time and the other columns are the absorbance at the wavelength specified in the column header. For the ELSD signal, the first column is the time and the second column is the signal from the detector.

Summary.xlsx is the Excel file that contains the retention time of each compound for all the experiments. The columns contain the following information: a unique identification for each line, a unique identification for each experiment (the data were collected with the same injection sequence), a true/false value to differentiate the QC data from the rest, a unique identification for the order of injection, the name of the compound, the collected retention time, the correction applied on the retention time of compounds detected with the ELSD, the corrected retention time, the date the sequence was started, the gradient time, the targeted pH value of the buffer, the pH value of the buffer measured before the analysis, a unique identification of the system used and a unique identification of the column used.

## 2 Experimental Design, Materials and Methods

### 2.1 Stock and working solutions

The different compounds were selected from the literature [1], [2], [3]. Each solution of single compound was independently prepared. Compounds were solubilized in water, methanol, or a mixture of both. When required, diluted formic acid or ammonia was added to help the solubilisation. Each stock solution was then diluted using water or a mixture of water and methanol to reach the targeted concentration. The targeted concentration was 20 µg·mL^−1^. A concentrated solution was requested for some compounds to detect them. For detailed information about each compound preparation, see Table S1.

The buffers consisted of commonly used volatile compounds (see Table 1). Such buffers at 10 mM were selected to be compatible with mass spectrometry detection.

### 2.2 Analytical method

The samples were injected on a Waters® XSelect HSS T3 100×2.1 mm 3.5 μm column (column volume of 350 µl) heated at 25 °C. An injection volume of 5 μL of diluted samples was used. The samples were analyzed with two different linear-gradient slopes with a flow rate of 0.3 ml·min^−1^ after equilibration of 120 min, corresponding to more than 100 times of the column volume. Details of each chromatographic method is given here under and summarized in Table 2. First, a linear gradient starts at 0% of methanol and 100% of buffer (φ_start_) and then increases to 95% methanol and 5% buffer (φ_end_) for 20 min. Then, the mobile phase composition is held for 5 min and goes back to the starting conditions in 1 min. The starting conditions are kept for 25 min to equilibrate the column with a mobile phase volume corresponding to more than 20 times of the column volume.

The 60 min linear gradient follows the same steps as the 20 min gradient one.

After the first replicates, all the injections were reproduced unless the retention time of the compound was not influenced by the pH. In those cases, only the pH 2 and 8 experimental conditions were replicated. In addition, the methods were adapted for the compounds with a noticeably low retention time. In order to reduce the experimental phase, two 4 min long gradient methods with slopes corresponding to the 20 and 60 min gradient described above have been used (see methods #3 and #4 in Table 2). The shortened method corresponding to 20 min gradient starts at 0% of methanol and reaches 19% of methanol in 4 min, returns to 0% of methanol in 1 min, and continues at that level for 12 min. The shortened method corresponding to 60 min gradient starts at 0% of methanol and reach 6.3% of methanol in 4 min, returns to 0% of methanol in 1 min and then holds for 6 min.

The five pH conditions were adapted with five different buffers. Their composition is described in Table 1.

### 2.3 Instrumentation

The analyses were performed on three different high-performance liquid chromatography (HPLC) systems described in Table 3. Two types of detectors were used: a UV-visible diode array detector (DAD) for compounds with chromophore and an evaporative light scattering detector (ELSD) for the remaining compounds. When the compounds were analysed on the DAD and the ELSD system, both detectors were connected in series (the ELSD is the last one). This configuration led to a delay of detection between both detectors caused by the length of the tubing connecting them. This delay of detection was corrected with the difference of retention time of a compound detected with both detectors such as the uracil (one of our QC compounds). The DAD was set to acquire spectra from 210 to 400 nm. The ELSD parameters were set at a gain of 2, a gas pressure of 40 PSI, a drift tube temperature of 50 °C and a nebulizer temperature of 75 °C. The dwell volume was determined following the method recommended by the equipment manufacturer [4].

This research was realized in an academic pharmaceutical quality control laboratory following the quality requirements from different regulatory authorities. The laboratory, which is GMP certified, is initiative-taking and has documented management of the risk. Each piece of equipment is maintained and qualified following standard operating procedures. Various parts of the equipment are periodically verified to ensure the reliable and consistent performance of the equipment.

More specifically, for this research, the HPLC systems were qualified every six months. The following list covers the different components of an HPLC system that were controlled:

• Flow rate accuracy.
• Gradient accuracy.
• Accuracy and linearity of the temperature of the autosampler and the column's oven.
• The general working state, the wavelength's precision, and the linearity of the UV detector.
• The accuracy of the injected volume and the repeatability and gain's linearity of the ELS detector.

Other systems, like analytical balance, the micropipettes, or the cold room, were also periodically qualified.

### 2.4 Technical validation

To ensure the reliability of the dataset, the first injection of each compound was performed individually to detect each compound regarding non-specific DAD and ELSD detection unambiguously.

A quality control (QC) sample composed of uracil, 3-cyanopyridine and ibuprofen was periodically injected to record their retention time through the different sequences and replicates. Those three molecules were selected based on their logP (−1.1, 0.2 and 3.5 respectively) to have low, middle, and high retention. They also have a high absorbance at a specific wavelength that makes them easily detectable and are stable at room temperature in solution. These QC data allowed to control the sequences and functioned as a system suitability test (SST). This QC sample of three molecules was injected at the beginning and the end of each injection sequence. The average retention time and the relative standard deviation (RSD) of the retention time of each compound in the QC sample are provided in Table 4. Using this QC sample multiple times during an injection sequence helped ensure that the analysis conditions were stable throughout the sequence and for all the replicates, the replicates were actual replicates in the same experimental conditions. Shared information related to the QC sample will facilitate the evaluation of the robustness of the dataset by its users. The limit of being inferior or equal to 5% for the RSD of each compound in the QC sample was fixed before starting the analyses. In Table 4, we can see that some values are a bit above the limit value for one of the QC compounds. Indeed, with the current setup of experiments comprising multiple sequences injected on different days with newly prepared buffers and multiple systems, the variability is defined as acceptable. No deviations are present in the dataset. The maximum RSD value computed is 5.4%, it occurs for the 3-cyanopyridine, which remains within the limit when rounded. The remaining QC compounds have maximum RSD values of 2.9%.

### 2.5 Usage notes

Considering the data were acquired on three different chromatographic systems, the future user might want to apply some correction to the retention times. For this, it is recommended to build transfer models between one of the systems, selected as the master system and each of the other two remaining systems. Those transfer models should be fitted on the QC's retention times. One transfer model should be created between each system for each condition.

### 2.6 Chemicals and reagents

Ammonium bicarbonate, ammonium acetate, ammonium formate, formic acid 99% and ammonia 25% were purchased from VWR Chemicals (Leuven, Belgium). Acetic acid was purchased from Merck Chemicals (Overijse, Belgium). Milli-Q water from a Merck milli-Q pump. Methanol HPLC gradient grade was purchased from J.T. Baker (Deventer, Netherlands).

Standard compounds: 2,2′-bipyridine, 2,2′-dinaphtyl ether, 2,3-dihydroxybenzoic acid, 2′,3′-dideoxyadenosine, 2′-deoxyguanosine hydrate, 3,4-dihydroxybenzoic acid, 3,5-dichlorophenol, 3-aminobenzoic acid, 3-cyanopyridine, 4-aminobenzoic acid, 4,4-aminophenol, 4-aminosalicylic acid, 4-hydroxybenzoic acid, 4-nitrophenol, acridone, adenine sulphate dihydrate, amitriptyline hydrochloride, L-(+)-arginine, L-aspartic acid, benzene, benzyl alcohol, betaxolol, biphenyl, carteolol, chlorobenzene, citric acid, coumarin, cytidine, cytosine, danthron (chrysazin), dibenzothiophene, dopamine (3-hydroxytyramine hydrochloride), dyphylline (7-(2,3-dihydroxypropyl-theophylline), ethylbenzene, etofylline (7-(2-hydroxyethyl-theophylline), eugenol, gallic acid hydrate, 4-gamma-aminobutyric acid, L-glutamic acid, glutaric acid, glycine, glycolic acid, hexylbenzene, hydroquinone, ibuprofen, imipramine, indole, indomethacin, lactic acid, L-(+)-lysine, DL-malic acid, DL-mandelic acid, mefenamic acid, 1-methyl-2-pyrrolidone, metoclopramide, miconazole nitrate, naphthalene, niacin (nicotinic acid), niacinamide (nicotinamide), papaverine, perphenazine, phenanthrene, 2-phenethylamine, phenol, phthalic acid, promethazine hydrochloride, quinoline, L‑serine, sulfamethazine, taurine (2-aminoethanesulfonic acid), tetracaine, thioridazine hydrochloride, thymine, toluene, L-(-)-tyrosine, uracil, uric acid, uridine, verapamil hydrochloride, xanthine from TCI; acetic acid; formic acid; D-(+)-glucose were purchased from VWR Chemicals (Leuven, Belgium). Chlordiazepoxide, chlorphenamine, oxazepam, salicylic acid from Fagron; benzoic acid, sodium thiosulfate, sulphate ion (sulfuric acid 95–97%) from Merck Chemicals (Overijse, Belgium). L-(+)-asparagine, procainamide hydrochloride 99%, sulphite from Acros Organics (Geelo, Belgium). Sodium nitrate, sodium nitrite, phenylacetic acid 99% from Alfa Aesar (Lancashire, United Kingdom). Beta-estradiol from Sigma; pindolol from Abcam (Rozenburg, Netherlands).

## Ethics Statement

The authors have nothing to declare. This work does not involve the use of human subjects, animal experiments, or data collected from social media platforms.

## CRediT authorship contribution statement

**Thomas Van Laethem:** Conceptualization, Methodology, Validation, Formal analysis, Investigation, Data curation, Writing – original draft, Visualization. **Priyanka Kumari:** Data curation, Writing – review & editing. **Philippe Hubert:** Conceptualization, Methodology, Resources, Writing – review & editing, Supervision, Funding acquisition. **Marianne Fillet:** Conceptualization, Methodology, Resources, Writing – review & editing, Supervision, Funding acquisition. **Pierre-Yves Sacré:** Conceptualization, Methodology, Resources, Writing – review & editing, Supervision, Project administration. **Cédric Hubert:** Conceptualization, Methodology, Resources, Writing – review & editing, Supervision, Project administration, Funding acquisition.

## Declaration of Competing Interest

The authors declare that they have no known competing financial interests or personal relationships that could have appeared to influence the work reported in this paper.

## Appendix Supplementary materials

Image, application 1

## Footnotes

Supplementary material associated with this article can be found in the online version at doi:10.1016/j.dib.2022.108017.
