# Supplementary material for: A pharmaceutical-related molecules dataset for reversed-phase chromatography retention time prediction built on combining pH and gradient time conditions
Source: Data Brief. 2022 Mar 4;42:108017. doi: 10.1016/j.dib.2022.108017 (PMC8931345; doi:10.1016/j.dib.2022.108017)
Supplement: Supplementary file 1 [file mmc1.docx]

# Supplementary materials

Table S1 - Solubilisation and dilution solutions of standard compounds

| Compound | Solubilisation solution | Dilution solution | Initial concentration [mg.ml^-1^] | Final concentration [mg.ml^-1^] | Detection mode |
| --- | --- | --- | --- | --- | --- |
| 2,2'-Bipyridine | MeOH | H_2_O | 1 | 0.02 | UV |
| 2,2'-Dinaphthyl Ether | MeOH | MeOH/H_2_O 50:50 | 1 | 0.02 | UV |
| 2,3-Dihydroxybenzoic acid | MeOH | H_2_O | 1 | 0.02 | UV |
| 2'-3'-Dideoxyadenosine | H_2_O | H_2_O | 1 | 0.02 | UV |
| 2'-Deoxyguanosine | H_2_O | H_2_O | 1 | 0.02 | UV |
| 3,4-Dihydroxybenzoic acid | MeOH | H_2_O | 1 | 0.02 | UV |
| 3,5-Dichlorophenol | MeOH | H_2_O | 1 | 0.02 | UV |
| 3-Aminobenzoic acid | H_2_O | H_2_O | 1 | 0.02 | UV |
| 3-Cyanopyridine | MeOH/H_2_O 30:70 | H_2_O | 1 | 0.02 | UV |
| 4-Aminobenzoic acid | H_2_O | H_2_O | 1 | 0.02 | UV |
| 4-Aminophenol | MeOH | H_2_O | 1 | 0.02 | UV |
| 4-Aminosalicylic acid | MeOH | H_2_O | 1 | 0.02 | UV |
| 4-Hydroxybenzoic acid | MeOH | H_2_O | 1 | 0.02 | UV |
| 4-Nitrophenol | MeOH | H_2_O | 1 | 0.02 | UV |
| Acetic acid | H_2_O | H_2_O | 2.1 | 0.21 | UV |
| Acridone | MeOH | MeOH/H_2_O 50:50 | 1 | 0.02 | UV |
| Adenine | H_2_O | H_2_O | 1 | 0.1 | UV |
| Amitriptyline | MeOH | H_2_O | 1 | 0.02 | UV |
| Asparagine | H_2_O | H_2_O | 1 | 0.2 | ELS |
| Benzene | MeOH | H_2_O | 1.76 | 0.0352 | UV |
| Benzoic acid | H_2_O/MeOH 1:2 | H_2_O | 1 | 0.02 | UV |
| Benzyl alcohol | H_2_O | H_2_O | 2.08 | 0.0416 | UV |
| Betaxolol | H_2_O | H_2_O | 1 | 0.02 | UV |
| Biphenyl | MeOH | H_2_O | 1 | 0.02 | UV |
| Carteolol | MeOH | H_2_O | 1 | 0.02 | UV |
| Chlordiazepoxide | MeOH | H_2_O | 1 | 0.02 | UV |
| Chlorobenzene | MeOH | H_2_O | 2.22 | 0.0888 | UV |
| Chlorphenamine | MeOH | H_2_O | 1 | 0.02 | UV |
| Citric acid | H_2_O | H_2_O | 1 | 0.2 | UV |
| Coumarin | MeOH | H_2_O | 1 | 0.02 | UV |
| Cytidine | H_2_O | H_2_O | 1 | 0.02 | UV |
| Cytosine | H_2_O | H_2_O | 1 | 0.2 | UV |
| Danthron | MeOH | H_2_O | 1 | 0.02 | UV |
| Dibenzothiophene | MeOH | H_2_O | 1 | 0.02 | UV |
| Dopamine | H_2_O | H_2_O | 1 | 0.02 | UV |
| Dyphylline | H_2_O | H_2_O | 1 | 0.1 | UV |
| Estradiol | MeOH | H_2_O | 1 | 0.02 | UV |
| Ethylbenzene | MeOH | H_2_O | 1.74 | 0.0348 | UV |
| Etofylline | H_2_O | H_2_O | 1 | 0.1 | UV |
| Eugenol | MeOH | H_2_O | 2.12 | 0.0424 | UV |
| Gallic acid | H_2_O | H_2_O | 1 | 0.2 | UV |
| Gamma-Aminobutyric acid | H_2_O | H_2_O | 1 | 0.2 | ELS |
| Glucose | H_2_O/MeOH 30:70 | H_2_O | 1 | 0.2 | UV |
| Glutamic acid | H_2_O | H_2_O | 1 | 0.2 | ELS |
| Glutaric acid | H_2_O | H_2_O | 1 | 0.2 | ELS |
| Glycine | H_2_O | H_2_O | 1 | 0.2 | ELS |
| Hexylbenzene | MeOH | H_2_O | 3.44 | 0.1376 | UV |
| Hydroquinone | H_2_O | H_2_O | 1 | 0.02 | UV |
| Ibuprofen | MeOH | H_2_O | 1 | 0.04 | UV |
| Imipramine | MeOH | H_2_O | 1 | 0.02 | UV |
| Indole | MeOH | H_2_O | 1 | 0.02 | UV |
| Indomethacin | MeOH | H_2_O | 1 | 0.02 | UV |
| Lactic acid | H_2_O | H_2_O | 2.4 | 0.24 | UV |
| L-Arginine | H_2_O | H_2_O | 1 | 0.2 | ELS |
| L-Aspartic acid | H_2_O | H_2_O | 1 | 0.2 | ELS |
| Lysine | H_2_O | H_2_O | 1 | 0.2 | ELS |
| Malic acid | H_2_O | H_2_O | 1 | 0.2 | ELS |
| Mandelic acid | H_2_O | H_2_O | 1 | 0.02 | UV |
| Mefenamic acid | MeOH | MeOH/H_2_O 50:50 | 1 | 0.02 | UV |
| Methylpyrrolidone | H_2_O | H_2_O | 2.06 | 0.0824 | UV |
| Metoclopramide | MeOH | H_2_O | 1 | 0.02 | UV |
| Miconazole | MeOH | H_2_O | 1 | 0.02 | UV |
| Naphthalene | MeOH | H_2_O | 1 | 0.02 | UV |
| Niacin | H_2_O | H_2_O | 1 | 0.02 | UV |
| Niacinamide | H_2_O | H_2_O | 1 | 0.02 | UV |
| Nitrate | H_2_O | H_2_O | 1 | 0.2 | UV |
| Nitrite | H_2_O | H_2_O | 1 | 0.2 | UV |
| Oxazepam | MeOH | H_2_O | 1 | 0.02 | UV |
| Papaverine | MeOH | H_2_O | 1 | 0.02 | UV |
| Perphenazine | MeOH | H_2_O | 1 | 0.02 | UV |
| Phenanthrene | MeOH | H_2_O | 1 | 0.02 | UV |
| Phenethylamine | H_2_O 0.2% formic acid | H_2_O | 1.92 | 0.0384 | UV |
| Phenol | H_2_O | H_2_O | 1 | 0.02 | UV |
| Phenylacetic acid | H_2_O | H_2_O | 1 | 0.02 | UV |
| Phthalic acid | MeOH | H_2_O | 1 | 0.02 | UV |
| Pindolol | H_2_O/MeOH 50:50 | H_2_O | 0.5 | 0.02 | UV |
| Procainamide | H_2_O | H_2_O | 1 | 0.02 | UV |
| Promethazine | MeOH | H_2_O | 1 | 0.02 | UV |
| Quinoline | MeOH | H_2_O | 2.18 | 0.0436 | UV |
| Salicylic acid | H_2_O | H_2_O | 1 | 0.2 | UV |
| Serine | H_2_O | H_2_O | 1 | 0.02 | UV |
| Sulfamethazine | MeOH | H_2_O | 1 | 0.02 | UV |
| Sulfate | H_2_O | H_2_O | 3.68 | 0.1472 | ELS |
| Sulfite | H_2_O | H_2_O | 1 | 0.2 | ELS |
| Taurine | H_2_O | H_2_O | 1 | 0.2 | ELS |
| Tetracaine | MeOH | H_2_O | 1 | 0.02 | UV |
| Thioridazine | H_2_O | H_2_O | 1 | 0.02 | UV |
| Thiosulfate | H_2_O | H_2_O | 1 | 0.2 | UV |
| Thymine | H_2_O | H_2_O | 1 | 0.02 | UV |
| Toluene | MeOH | H_2_O | 1.74 | 0.0348 | UV |
| Tyrosine | H_2_O | H_2_O | 0.2 | 0.02 | UV |
| Uracil | H_2_O | H_2_O | 0.5 | 0.02 | UV |
| Uric acid | H_2_O + 1 drop ammonia 25% | H_2_O | 0.5 | 0.02 | UV |
| Uridine | H_2_O | H_2_O | 1 | 0.2 | UV |
| Verapamil | H_2_O | H_2_O | 1 | 0.02 | UV |
| Xanthine | H_2_O + 1 drop ammonia 25% | H_2_O | 1 | 0.1 | UV |
